# Supplementary material for: Wrist Movement Variability Assessment in Individuals with Parkinson’s Disease
Source: Healthcare (Basel). 2022 Aug 30;10(9):1656. doi: 10.3390/healthcare10091656 (PMC9498573; doi:10.3390/healthcare10091656)
Supplement: Supplementary file 1 [file healthcare-10-01656-s001.zip › healthcare-1862256-supplementary.pdf]

Table S1. Variables that contribute the most to data variability description.

Variable	Description
T6F2S1	root mean square of the capacitive signal during wrist extension in regular speed detected by sensor 1, on the radial-ulnar - y axis.
T10F2S1	root mean square of the capacitive signal during wrist extension in fast speed detected by sensor 1, on the radial-ulnar - y axis.
T10F4S1	mean of the absolute values of the first differences of normalized capacitive signal during wrist extension in fast speed on detected by sensor 1, on the radial-ulnar - y axis.
T6F3S1	mean of the absolute values of the first differences of capacitive signal during wrist extension in regular speed on detected by sensor 1, on the radial-ulnar - y axis.
T10F1S1	mean absolute value of the capacitive signal during wrist extension in fast speed detected by sensor 1, on the radial-ulnar - y axis.
T6F5S1	mean of the absolute values of the second differences of capacitive signal during wrist extension in regular speed detected by sensor 1, on the radial-ulnar - y axis.
T6F4S1	mean of the absolute values of the first differences of normalized capacitive signal during wrist extension in regular speed detected by sensor 1, on the radial-ulnar - y axis.
T10F2S2	root mean square of capacitive signal during wrist extension in fast speed detected by sensor 2, on the proximal-distal - z axis.
T5F2S2	root mean square of capacitive signal during wrist flexion in regular speed detected by sensor 2, on the proximal-distal - z axis.
T9F2S2	root mean square of capacitive signal during wrist flexion in fast speed detected by sensor 2, on the proximal-distal - z axis.
T6F2S2	root mean square of capacitive signal during wrist extension in regular speed detected by sensor 2, on the proximal-distal - z axis.
T6F1S2	mean absolute value of the capacitive signal during wrist extension in regular speed detected by sensor 2, on the proximal-distal - z axis.
T6F5S2	mean of the absolute values of the second differences of capacitive signal during wrist extension in regular speed detected by sensor 2, on the proximal-distal - z axis.
T6F3S2	mean of the absolute values of the first differences of capacitive signal during wrist extension in regular speed detected by sensor 2, on the proximal-distal - z axis.
